# Neoadjuvant PF-05280014 (a potential trastuzumab biosimilar) versus trastuzumab for operable HER2+ breast cancer

**DOI:** 10.1038/s41416-018-0147-1

**Published:** 2018-07-13

**Authors:** Philip E. Lammers, Magdolna Dank, Riccardo Masetti, Richat Abbas, Fiona Hilton, Jennifer Coppola, Ira Jacobs

**Affiliations:** 1 Meharry Medical College, 1005 Dr. D.B. Todd Jr. Blvd, Nashville, TN 37208-3501 USA; 2 Semmelweis University, Cancer Center, Tömő u. 25-29, Budapest, 1083 Hungary; 3 Catholic University of Rome, Largo Agostino Gemelli, 8, Rome, 00168 Italy; 4 Pfizer Inc., 500 Arcola Road, Collegeville, PA 19426 USA; 5 Pfizer Inc., 450 Eastern Point Road, Groton, CT 06340 USA; 6 Pfizer Inc., 235 and 219 East 42nd Street, New York, NY 10017-5755 USA

## Abstract

**Background:**

This randomised, double-blind study compared pharmacokinetics, efficacy, safety and immunogenicity of PF-05280014 (potential trastuzumab biosimilar) and trastuzumab reference product (Herceptin) sourced from the European Union (trastuzumab-EU) as neoadjuvant treatment for operable human epidermal growth factor receptor 2 (HER2)-positive breast cancer.

**Methods:**

Patients (*N* = 226), stratified by primary tumour size and hormone receptor status, were randomised 1:1 to PF-05280014 or trastuzumab-EU (8 mg/kg loading dose; 6 mg/kg thereafter), each with docetaxel and carboplatin, every 3 weeks for six treatment cycles. Primary endpoint was percentage of patients with trough plasma concentration (*C*_trough_) >20 μg/ml at Cycle 5 (Cycle 6 predose). Efficacy endpoints included pathological complete response and objective response rate. Non-inferiority of PF-05280014 to trastuzumab-EU was declared if the lower limit of the 95% confidence interval for the stratified difference between groups in the percentage of patients with Cycle 5 *C*_trough_ >20 μg/ml was above the prespecified non-inferiority margin of – 12.5%.

**Results:**

For PF-05280014 vs trastuzumab-EU patients, respectively, 92.1% vs 93.3% had Cycle 5 *C*_trough_ >20 μg/ml; the lower limit of the 95% confidence interval (− 8.02%, 6.49%) for the stratified difference between groups was above the non-inferiority margin (– 12.5%). Pathological complete response (47.0% vs 50.0%) and central radiology review-assessed objective response (88.1% vs 82.0%) rates were comparable. Incidence of all-causality, grade 3–4 treatment-emergent adverse events was 38.1% vs 45.5%; antidrug antibody rates were 0% vs 0.89%.

**Conclusions:**

PF-05280014 demonstrated non-inferior pharmacokinetics and comparable efficacy, safety and immunogenicity to trastuzumab-EU in patients with operable HER2-positive breast cancer receiving neoadjuvant chemotherapy.

## Introduction

Trastuzumab is a recombinant humanised immunoglobulin G1 monoclonal antibody that targets human epidermal growth factor receptor 2 (HER2) and is approved for the treatment of HER2-overexpressing breast and gastric cancers.^1,2^ HER2 overexpression occurs in 15–20% of invasive breast cancers; it is associated with more-aggressive biological behaviour and, in the absence of treatment with HER2-targeted therapy, worse clinical outcomes.^3–6^ The addition of trastuzumab to adjuvant chemotherapy for HER2-positive (HER2+) early breast cancer or to chemotherapy for HER2+ metastatic disease reduces the risk for recurrence or disease progression and prolongs survival as compared with chemotherapy alone.^7–10^ When added to neoadjuvant chemotherapy, trastuzumab improves rates of event-free survival and pathological complete response (pCR) over chemotherapy alone.^11,12^

Despite the clinical benefits associated with HER2-directed therapy, physicians worldwide often encounter barriers to prescribing trastuzumab, leading to less than optimal treatment of patients with HER2+ breast cancer.^13,14^ The availability of biosimilars may expand access to biologic therapies such as trastuzumab and provide patients with additional safe and efficacious treatment options. Biosimilars are biologic drugs that are highly similar to a licensed (i.e., originator or reference) biologic product.^15–17^ To receive regulatory approval, a proposed biosimilar product must show no clinically meaningful differences in safety, purity or potency compared with the originator biologic, based on the totality of the evidence obtained from comparative assessments of the two products.^15–17^ Regulatory agencies recommend a stepwise approach to generating these data that begins with comprehensive analytical (i.e., structural and functional) characterisation followed by non-clinical testing and culminates with a comparative clinical study (or studies) to confirm similarity between the proposed biosimilar and originator product in pharmacokinetics, efficacy, safety and immunogenicity.^15–17^

PF-05280014 is under development as a potential biosimilar of trastuzumab (Herceptin). Comparative non-clinical assessments of PF-05280014 and trastuzumab reference products marketed in the European Union (trastuzumab-EU; Herceptin, Roche Registration GmbH, Grenzach-Wyhlen, Germany) and United States (trastuzumab-US; Herceptin, South San Francisco, CA) demonstrated PF-05280014 has the same primary amino-acid sequence as the licensed trastuzumab, with similar in vitro functional properties and in vivo pharmacokinetics, antidrug antibody (ADA) responses and tolerability.^18^ Furthermore, two single-dose comparability studies conducted in healthy male volunteers demonstrated PF-05280014, trastuzumab-EU and trastuzumab-US have similar pharmacokinetics, safety and immunogenicity profiles.^19,20^ An additional, distinct comparative safety and efficacy study (NCT01989676) is evaluating PF-05280014 versus trastuzumab-EU, each administered in combination with paclitaxel, as first-line treatment for patients with HER2+ metastatic breast cancer.^21^

We report the results of a comparative clinical trial conducted to compare pharmacokinetics, efficacy, safety and immunogenicity of PF-05280014 versus trastuzumab-EU, each administered in combination with docetaxel and carboplatin, as neoadjuvant treatment for patients with operable HER2+ breast cancer.^22^ It was hypothesised that PF-05280014 was non-inferior to trastuzumab-EU, based on pharmacokinetics data.

## Methods

### Study population

Eligible patients were women aged 18 years or older with histologically confirmed invasive breast cancer that exhibited HER2 gene amplification by fluorescent in situ hybridisation, chromogenic in situ hybridisation or dual in situ hybridisation, as defined by the manufacturer’s kit instruction; or HER2 overexpression by immunohistochemistry (IHC) categorised as IHC3+; or HER2 overexpression by IHC categorised as IHC2+ with fluorescent, chromogenic, or dual in situ hybridisation confirmation. HER2+ tumour status was determined by the site at the time of diagnosis, using either a sponsor-approved assay or two different analytical test methods that were not considered sponsor-approved but both demonstrated unequivocal (i.e., IHC3+) results, and confirmed retrospectively by the sponsor-provided central laboratory. If tumour HER2 status could not be determined via local testing, it was evaluated by central laboratory assessment.

Patients with measurable disease (longest diameter ≥ 2.0 cm) in the breast after diagnostic biopsy and known hormone (oestrogen and progesterone) receptor status at study entry were included. For patients with unknown hormone receptor status, oestrogen and progesterone receptor status testing was performed at screening, via local or central laboratory assessment. Oestrogen and progesterone receptor positivity was determined by local site guidelines based on accepted standards. Baseline tumour assessments were performed within 6 weeks prior to randomisation and included computed tomography (CT), or magnetic resonance imaging of the chest if a CT scan could not be performed, and bilateral mammography or ultrasound of the breast. Patients were planned to undergo definitive surgical resection of breast tumour (i.e., lumpectomy or mastectomy with sentinel node biopsy or axillary lymph node dissection) and neoadjuvant chemotherapy. Other inclusion criteria included: Eastern Cooperative Oncology Group performance status 0–1; left ventricular ejection fraction (LVEF) ≥ 55% as measured by two-dimensional echocardiogram (ECHO) or multi-gated acquisition scan (MUGA); and normal laboratory values.

Key exclusion criteria included bilateral breast cancer; inflammatory breast cancer; presence of known distant metastases, as determined by the investigator; prior chemotherapy, endocrine therapy, biologic therapy, radiation or surgery, except diagnostic biopsy for primary breast cancer; other concomitant active malignancy or history of malignancy in the past 5 years, except treated basal cell carcinoma of the skin or carcinoma in situ of the cervix; history of documented or current congestive heart failure; current high-risk uncontrolled arrhythmias; angina pectoris requiring treatment; clinically significant valvular disease; evidence of transmural infarction on electrocardiogram; or poorly controlled hypertension.

### Study design, procedures and treatments

This was an international, double-blind, randomised clinical trial initiated at 67 sites in 10 countries across Europe (EudraCT registration number 2013-004679-11) and in the United States (ClinicalTrials.gov identifier NCT02187744).^22,23^

**Fig. 1:**
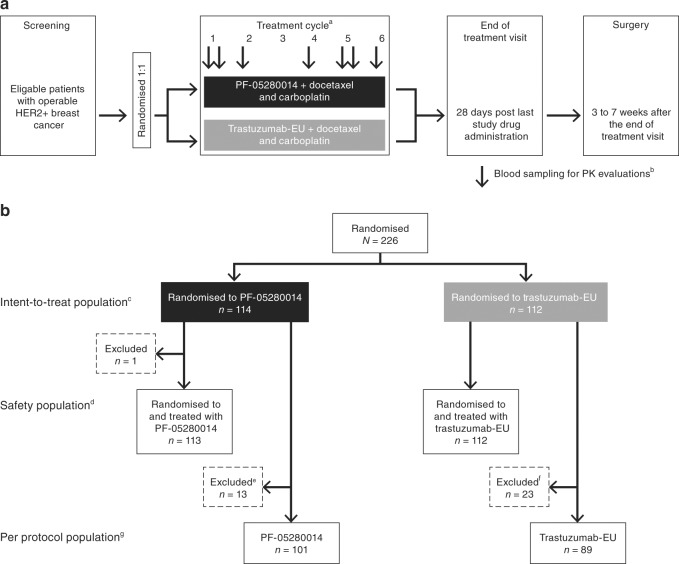
Overall study design and disposition of patients. **a** Study design and **b** patient disposition. ^a^On Day 1 Cycle 1, patients received a loading dose (8 mg/kg) of PF-05280014 or trastuzumab-EU infused over 90 min followed by docetaxel (75 mg/m^2^; 60-min intravenous infusion) and carboplatin (target AUC: 6; ≥ 15-min intravenous infusion). Subsequent infusions of PF-05280014 or trastuzumab-EU (6 mg/kg, over 30 to 90 min), docetaxel and carboplatin were administered every 3 weeks for a total of six treatment cycles. ^b^Blood samples were collected predose (−2.5 h to −5 min prior to infusion) on Day 1 of Cycles 1, 2, 4, 5 and 6, and at 1-h post dose on Day 1 of Cycles 1 and 5 for determination of PF-05280014 or trastuzumab-EU serum concentrations. ^c^The intent-to-treat population consisted of all patients randomised to PF-05280014 or trastuzumab-EU. ^d^The safety population comprised all patients who received at least one dose of study drug. ^e^Patients in the PF-05280014 group were excluded from the per protocol population for the following reasons: Cycle 5 trough sample taken outside protocol-specified window (*n* = 6, 46.2%), fewer than six cycles of trastuzumab (*n* = 5, 38.5%), no Cycle 5 trough pharmacokinetic sample (*n* = 2, 15.4%), and trastuzumab treatment delay > 1 week (*n* = 1, 7.7%). A patient may have met multiple criteria for exclusion and may have been counted more than once. ^f^Patients in the trastuzumab-EU group were excluded from the per protocol population for the following reasons: Cycle 5 trough sample taken outside protocol-specified window (*n* = 13, 56.5%), fewer than six cycles of trastuzumab (*n* = 5, 21.7%), trastuzumab treatment delay > 1 week (*n* = 3, 13.0%), Cycle 5 trough sample taken post dose (*n* = 1, 4.3%), no lesion > 2 cm in breast (*n* = 1, 4.3%), and missing HER2 sample (*n* = 1, 4.3%). A patient may have met multiple criteria for exclusion and may have been counted more than once. ^g^The per protocol population consisted of all randomised patients who received six cycles of PF-05280014 or trastuzumab-EU and had no temporary delays in treatment lasting > 1 week or other significant protocol deviations. AUC = area under the curve; HER2 = human epidermal growth factor receptor 2; trastuzumab-EU = licensed trastuzumab sourced from the European Union

Patients who satisfied the eligibility criteria were stratified by primary tumour size (< 5 cm vs ≥ 5 cm) and hormone receptor status (positive vs negative) and randomised 1:1 to receive PF-05280014 or trastuzumab-EU (Herceptin), each given in combination with docetaxel and carboplatin (Fig. 1a). On Day 1 Cycle 1, patients received a loading dose (8 mg/kg, over 90-min intravenous (IV) infusion) of PF-05280014 or trastuzumab-EU followed by docetaxel (75 mg/m^2^; 60-min IV infusion) and carboplatin (target area under the curve: 6; ≥ 15-min IV infusion). Subsequent infusions of PF-05280014 or trastuzumab-EU (6 mg/kg, over 30 to 90 min), docetaxel and carboplatin were administered every 3 weeks for six treatment cycles. Neoadjuvant therapy with trastuzumab in combination with docetaxel and carboplatin has demonstrated efficacy and safety in patients with HER2+ early breast cancer.^24^ Furthermore, the trastuzumab treatment regimen used in this study is consistent with trastuzumab-EU product labelling and is in line with the National Comprehensive Cancer Network recommendations on the preferred preoperative/adjuvant therapy regimens for HER2+ breast cancer.^2,25^ Patients who experienced toxicity attributed to PF-05280014 or trastuzumab-EU were required to temporarily or permanently discontinue trastuzumab treatment; dose reductions were not permitted. Granulocyte colony-stimulating factor was used for prophylactic or therapeutic management of haematologic toxicities attributed to docetaxel. Reductions in the docetaxel dose or discontinuation of treatment with docetaxel were permitted.

Patients had a follow-up assessment, i.e., end of treatment (EOT) visit, 28 (±7) days after final study drug administration and underwent definitive surgical resection of their primary breast tumour within 3–7 weeks following the EOT visit. Systemic therapy given after resection was not mandated by the protocol and PF-05280014 or trastuzumab-EU was not provided for treatment in the adjuvant setting. Patients were not followed for clinical endpoints after resection.

### Objectives and endpoints

The primary objective was to determine whether PF-05280014 is non-inferior to trastuzumab-EU, based on pharmacokinetics data. The mechanism of action of trastuzumab is mediated through its binding to the target receptor HER2, and full receptor saturation is expected to drive efficacy of trastuzumab.^1,2,26^ Accordingly, similar trough plasma concentrations (*C*_trough_) for PF-05280014 and trastuzumab-EU would support similar efficacy between treatments. Furthermore, *C*_trough_ > 20 μg/ml is a therapeutic target threshold concentration for trastuzumab and has been used to demonstrate noninferiority of subcutaneous trastuzumab to intravenous trastuzumab in the neoadjuvant setting.^26^ Therefore, non-inferiority was assessed by comparing the percentage of patients with Cycle 5 *C*_trough_ (Cycle 6 predose) > 20 μg/ml (primary endpoint) in the PF-05280014 group with that in the trastuzumab-EU group. This time point (Cycle 5 *C*_trough_) was selected because it would be expected to reflect steady state drug concentration based on population predicted pharmacokinetic exposure values for the approved 3-weekly dosing regimen used in this study.^2^

Secondary objectives were to evaluate the efficacy, safety, immunogenicity and other pharmacokinetic measures of PF-05280014 and trastuzumab-EU. Efficacy was measured by the percentage of patients with pCR, defined as the absence of invasive neoplastic cells in the breast and lymph nodes following surgery after treatment completion (remaining ductal carcinoma in situ was accepted),^26^ and objective response rate (ORR), defined as the percentage of patients who had complete or partial response at Cycle 6/EOT. Safety was characterised by the type, incidence, severity, timing, seriousness and relatedness of adverse events (AEs) to study drug, including cardiotoxicity, signs and symptoms of anaphylaxis, infusion-related reactions and laboratory abnormalities. Immunogenicity was measured by the incidence of ADAs, including neutralising antibodies (NAbs). Pharmacokinetics was further assessed by measuring *C*_trough_ at Cycles 1 (Cycle 2 predose), 3 (Cycle 4 predose) and 4 (Cycle 5 predose), as well as *C*_max_ (maximum drug concentration, 1 h post dose) at Cycles 1 and 5.

### Assessments

Blood samples for determining PF-05280014 or trastuzumab-EU serum concentrations were collected predose (–2.5 h to –5 min prior to infusion) on Day 1 of Cycles 1, 2, 4, 5 and 6, and at 1-h post dose on Day 1 of Cycles 1 and 5. Samples were analysed using a validated enzyme-linked immunosorbent assay with a lower limit of quantification of 0.500 µg/ml. Pathological response status was determined by an investigator-designated qualified pathologist. Tumour assessments were performed at screening, end of Cycle 3, EOT and as clinically needed. Objective response status was determined by a central radiology laboratory and by the investigators using Response Evaluation Criteria in Solid Tumours, version 1.1. Central radiology assessments were used to calculate ORR at Cycle 6/EOT.

AEs were monitored continuously from the time the patient received at least one dose of study drug through the last patient visit. The severity of AEs was graded in accordance with the National Cancer Institute Common Terminology Criteria for Adverse Events, version 4.03. Cardiac monitoring (MUGA scan or ECHO) was performed at screening, end of Cycle 3, EOT visit and as clinically indicated. Serum samples for detecting ADAs and NAbs were collected on Day 1 (predose) of Cycles 1, 2, 4 and 6 (Fig. 1a), and during the EOT visit. Samples were tested for ADAs following a tiered approach of screening, confirmation and titre. Samples were first analysed using a validated electrochemiluminescent immunoassay specific to PF-05280014 or trastuzumab-EU. ADA-positive samples were then analysed for cross-reactivity using the alternate assay. Confirmed ADA-positive samples were tested for NAb using a validated electrochemiluminescent immunoassay specific to the monoclonal antibody administered; NAb-positive samples were then analysed for cross-reactivity using the alternate assay.

### Statistical methods

This study tested the hypothesis that the percentage of patients exhibiting Cycle 5 *C*_trough_ (Cycle 6 predose) > 20 μg/ml in the PF-05280014 group was non-inferior to that in the trastuzumab-EU group, using a margin of – 12.5%. This margin was selected based on clinical considerations rather than statistical considerations (e.g., a meta-analysis of previous data) as there is little previous evidence in the neoadjuvant setting outside of the HannaH trial, which evaluated non-inferiority of subcutaneous trastuzumab to intravenous trastuzumab based on pCR rate and using a margin of − 12.5%.^26^ A minimum of 188 patients (*n* = 94/arm) was required to provide 85% power to test for non-inferiority in the primary analysis. Considering a possible 15% attrition rate, a total sample size of ~ 220 patients (*n* = 110/arm) was planned to ensure the required minimum number of patients for the non-inferiority analysis.

Differences between groups in the percentage of patients with Cycle 5 *C*_trough_ (Cycle 6 predose) > 20 μg/ml, the percentage of patients who had pCR and the ORR were estimated, along with corresponding 95% confidence intervals, using the normal approximation to the binomial distribution and adjusting for randomisation strata. If the lower limit of the CI for the primary endpoint was above – 12.5%, the null hypothesis was rejected and PF-05280014 was considered non-inferior to trastuzumab-EU. Cycle 5 *C*_trough_ (Cycle 6 predose) values were log-transformed and the geometric ratio (PF-05280014 to trastuzumab-EU) of the means and corresponding 95% CI were also estimated (secondary pharmacokinetics endpoint).

Pharmacokinetics and efficacy analyses were performed in the per protocol population, defined as all randomised patients who received six cycles of PF-05280014 or trastuzumab-EU and had no temporary delays in treatment that lasted > 1 week and no other significant protocol deviations. Sensitivity analyses of the primary and efficacy endpoints were performed in the intent-to-treat population (i.e., all randomised patients). Safety analyses, including AE, ADA and NAb analyses, were performed using the safety population (i.e., all patients who received at least one dose of study drug).

## Results

### Patient disposition, demographics and baseline characteristics

A total of 226 patients were randomised to PF-05280014 (*n* = 114) or trastuzumab-EU (*n* = 112) and were included in the intent-to-treat population. Of these, one patient was randomised to but did not receive treatment with PF-05280014; the remaining 225 patients received study treatment as assigned and were included in the safety population (Fig. 1b). Of the 226 randomised patients, 190 (PF-05280014, *n* = 101; trastuzumab-EU, *n* = 89) met the requirements for the per protocol population, and 36 (PF-05280014, *n* = 13; trastuzumab-EU, *n* = 23) were excluded for one or more of the following reasons, which were determined prior to breaking the blind (Fig. 1b): Cycle 5 trough sample taken outside the protocol-specified window, Cycle 5 trough sample taken post dose, fewer than six cycles of trastuzumab treatment, no Cycle 5 trough pharmacokinetics sample, no lesion > 2 cm in breast, missing HER2 sample and/or trastuzumab treatment delay > 1 week. Patient demographics and other baseline characteristics for the intent-to-treat population were comparable between treatment groups (Table 1).

**Table 1:** Baseline demographics (intent-to-treat population)^a^

	PF-05280014 (*n* = 114)	Trastuzumab-EU (*n* = 112)	Total (*N* = 226)
Age, mean (±SD), years	54.0 (11.9)	51.2 (12.7)	52.6 (12.3)
Race, *n* (%)			
White	112 (98.2)	109 (97.3)	221 (97.8)
Black	1 (0.9)	0	1 (0.4)
Asian	1 (0.9)	3 (2.7)	4 (1.8)
Ethnicity, *n* (%)			
Hispanic/Latino	0	1 (0.9)	1 (0.4)
Not Hispanic/Latino	114 (100.0)	111 (99.1)	225 (99.6)
Mean body mass index (±SD), kg/m^2^	28.2 (5.9)	27.7 (6.2)	27.9 (6.1)
Primary tumour size, *n* (%)			
<5 cm	89 (78.1)	89 (79.5)	178 (78.8)
≥5 cm	25 (21.9)	23 (20.5)	48 (21.2)
Oestrogen receptor status, *n* (%)			
Positive	58 (50.9)	54 (48.2)	112 (49.6)
Negative	56 (49.1)	58 (51.8)	114 (50.4)
Progesterone receptor status, *n* (%)			
Positive	41 (36.0)	40 (35.7)	81 (35.8)
Negative	73 (64.0)	72 (64.3)	145 (64.2)

^a^Baseline was defined as the value recorded at Day 1 Cycle 1. If this value was missing, the value recorded at screening was used.

Trastuzumab-EU = licensed trastuzumab sourced from the European Union; SD = standard deviation

### Pharmacokinetic analyses

In an analysis of the primary endpoint using the per protocol population, 93 (92.1%) patients treated with PF-05280014 and 83 (93.3%) patients treated with trastuzumab-EU exhibited Cycle 5 *C*_trough_ (Cycle 6 predose) >20 μg/ml (Table 2). The stratified estimated difference between PF-05280014 and trastuzumab-EU was – 0.76%, and the lower limit of the 95% CI (– 8.02%, 6.49%) was above the non-inferiority margin of – 12.5%. In an analysis of secondary pharmacokinetics endpoints using the per protocol population, the geometric mean *C*_trough_ at Cycle 5 was 34.59 and 34.56 µg/ml for PF-05280014 and trastuzumab-EU, respectively; the PF-05280014 to trastuzumab-EU ratio was 100.06% (95% CI: 81.5%, 122.9%). In addition, at each study cycle, trastuzumab serum concentrations appeared comparable between PF-05280014 and trastuzumab-EU (Supplementary Tables S1 and S2).

**Table 2:** Primary pharmacokinetics analysis of patients reporting Cycle 5 *C*_trough_^a^ >20 μg/ml (per protocol population)

	PF-05280014 (*n* = 101)	Trastuzumab-EU (*n* = 89)
Patients with Cycle 5 *C*_trough_^a^ > 20 μg/ml, % (95% CI)	92.1 (85.0, 96.5)	93.3 (85.9, 97.5)
Stratified difference between PF-05280014 and trastuzumab-EU^b^	−0.76	
Standard error for the difference	3.70	
95% CI (stratified) for the difference	−8.02, 6.49	

^a^Cycle 6 predose.

^b^Stratified analysis was based on the normal approximation to the binomial distribution, adjusting for randomisation strata (primary tumour size < 5 cm vs ≥5  cm; oestrogen receptor-positive vs oestrogen receptor-negative; and progesterone receptor-positive vs progesterone receptor-negative).

CI = confidence interval; *C*_trough_ = trough plasma concentration; trastuzumab-EU = licensed trastuzumab sourced from the European Union

### Efficacy analyses

Pathologic response and overall tumour response assessments for the per protocol population are summarised in Table 3. Of the patients in the PF-05280014 (*n* = 100) and trastuzumab-EU (*n* = 86) groups who had surgery, 47.0% (95% CI: 36.9%, 57.2%) and 50.0% (95% CI: 39.0%, 61.0%), respectively, had a pCR; the stratified estimated difference between groups was – 2.81% (95% CI: – 16.58%, 10.96%). The ORR at Cycle 6/EOT was 88.1% (95% CI: 80.2%, 93.7%) for PF-05280014 and 82.0% (95% CI: 72.5%, 89.4%) for trastuzumab-EU, based on central radiology assessments; the stratified estimated difference between groups was 5.96% (95% CI: –4.01%, 15.94%).

**Table 3:** Pathological response and overall tumour response assessments (per protocol population)

	PF-05280014 (*n* = 101)	Trastuzumab-EU (*n* = 89)
Pathological response assessment		
Response category, *n* (%)		
pCR	47 (46.5)	43 (48.3)
pPR	51 (50.5)	40 (44.9)
No pathological response	2 (2.0)	3 (3.4)
Not done^a^	1 (1.0)	3 (3.4)
Patients who had surgery		
*n* (%)	100 (99.0)	86 (96.6)
Patients with pCR,^b^ *n* (%)	47 (47.0)	43 (50.0)
95% CI	36.9, 57.2	39.0, 61.0
Stratified difference in pCR between PF-05280014 and trastuzumab-EU^c^	−2.81	
Standard error for the difference	7.03	
95% CI (stratified) for the difference	−16.58, 10.96	
Overall response assessment (per central radiology review)		
Overall response category at Cycle 6/EOT, *n* (%)		
Complete response	3 (3.0)	0
Partial response	86 (85.1)	73 (82.0)
Stable disease	7 (6.9)	4 (4.5)
Progressive disease	2 (2.0)	1 (1.1)
Non-evaluable	1 (1.0)	6 (6.7)
Non-complete response/non-progressive disease	1 (1.0)	3 (3.4)
Missing	1 (1.0)	2 (2.2)
ORR^d^		
*n* (%)	89 (88.1)	73 (82.0)
95% CI	80.2, 93.7	72.5, 89.4
Stratified difference in ORR between PF-05280014 and trastuzumab-EU^c^	5.96	
Standard error for the difference	5.09	
95% CI (stratified) for the difference	−4.01, 15.94	

^a^Pathology data were not recorded or response was not assessed for the following reasons: completed the study but had no surgery (PF-05280014, *n* = 1; trastuzumab-EU, *n* = 1) or completed treatment but lost to follow-up prior to surgery (trastuzumab-EU, *n* = 2).

^b^The denominators for percentages of patients with pCR included only patients who had surgery.

^c^Stratified analysis was based on the normal approximation to the binomial distribution, adjusting for randomisation strata (primary tumour size < 5 cm vs ≥ 5 cm; oestrogen receptor-positive vs oestrogen receptor-negative; and progesterone receptor-positive vs progesterone receptor-negative).

^d^ORR was defined as the percentage of patients within each treatment group who achieved complete response or partial response by Cycle 6/EOT, in accordance with Response Evaluation Criteria in Solid Tumours, version 1.1.

CI = confidence interval; EOT = end of treatment; ORR = objective response rate; pCR = pathological complete response; pPR = pathological partial response; trastuzumab-EU = licensed trastuzumab sourced from the European Union

### Safety

Among patients included in the safety population (PF-05280014, *n* = 113; trastuzumab-EU, *n* = 112), 109 (96.5%) in the PF-05280014 group and 109 (97.3%) in the trastuzumab-EU group received six cycles of trastuzumab. Six patients each in the PF-05280014 (5.3%) and trastuzumab-EU (5.4%) groups had a delay in trastuzumab treatment. Eight (7.1%) patients administered PF-05280014 and 7 (6.3%) who received trastuzumab-EU had an interrupted trastuzumab infusion.

The majority of all patients experienced at least one treatment-emergent AE (TEAE) owing to any cause, with a total of 569 events reported by 109 (96.5%) patients in the PF-05280014 group and 511 events reported by 106 (94.6%) patients in the trastuzumab-EU group (Table 4). The incidence of TEAEs and serious TEAEs was comparable between groups. The TEAEs most frequently reported by patients (*n* (%)) in the PF-05280014 and trastuzumab-EU groups, respectively, were alopecia (72 (63.7%) and 69 (61.6%)), anaemia (56 (49.6%) and 51 (45.5%)) and neutropaenia (38 (33.6%) and 41 (36.6%)). Grade 3–4 TEAEs were reported in 43 (38.1%) and 51 (45.5%) patients in the PF-05280014 and trastuzumab-EU groups, respectively. Seven (6.2%) patients treated with PF-05280014 experienced seven serious AEs (SAEs; febrile neutropaenia, neutropaenia, pancytopenia, proctitis, device-related sepsis, injection-site abscess and increased blood creatinine); six (5.4%) patients treated with trastuzumab-EU experienced 10 SAEs (anaemia, febrile neutropaenia (*n* = 2), neutropaenia (two SAEs, *n* = 1), gastrointestinal infection, tooth infection, hip fracture, dehydration and hypokalaemia).

**Table 4:** All-causality, treatment-emergent adverse events (safety population)^a^

	PF-05280014 (*n* = 113)	Trastuzumab-EU (*n* = 112)
Number of AEs	569	511
Patients with event, *n* (%)		
AEs	109 (96.5)	106 (94.6)
SAEs^b^	7 (6.2)	6 (5.4)
Grade 3 or 4 AEs	43 (38.1)	51 (45.5)
Grade 5 AEs	1 (0.9)	0
Discontinued study due to AEs	1 (0.9)	3 (2.7)
Discontinued from any treatment^c^ due to AEs	4 (3.5)	3 (2.7)
Dose reduced or temporarily discontinued for any treatment^c^ due to AEs	37 (32.7)	30 (26.8)

^a^Includes data up to 50 days after the last dose of study drug. Patients were counted only once per treatment in each row, except for the Number of AEs.

^b^As determined by investigator.

^c^Trastuzumab (PF-05280014 or trastuzumab-EU), docetaxel or carboplatin.

AE = adverse event; SAE = serious adverse event; trastuzumab-EU = licensed trastuzumab sourced from the European Union

No TEAEs indicative of infusion-related reactions were reported in the PF-05280014 group; two (1.8%) patients in the trastuzumab-EU group experienced non-serious events of pyrexia and tachypnea. Four (3.5%) and three (2.7%) patients in the PF-05280014 and trastuzumab-EU groups, respectively, permanently discontinued from any treatment due to AEs during the treatment period. One patient in the PF-05280014 group died due to a treatment-related SAE of pancytopenia; no other patients died during the study. No TEAEs of congestive heart failure or clinically significant LVEF abnormalities were reported by patients in either treatment group. There were no notable differences between the treatment groups in mean LVEF results (Supplementary Table S3). One (0.88%) and 10 (8.93%) patients in the PF-05280014 and trastuzumab-EU groups, respectively, had a decline in LVEF of ≥ 10% from baseline. However, no patient in either group had LVEF < 53% at any time point measured. Furthermore, no individual abnormalities or shifts in laboratory values were considered clinically relevant.

### Immunogenicity

No patients in the PF-05280014 group and one (0.89%) patient in the trastuzumab-EU group had positive ADA titres (Table 5). The single positive ADA titre of 2.39 was recorded at predose Cycle 1. This patient also recorded a cross-reactivity ADA assay titre of 2.58, but was negative in all subsequent ADA tests and also tested negative for NAbs.

**Table 5:** Incidence of ADAs by visit and overall (safety population)^a^

Visit	ADA status	PF-05280014	Trastuzumab-EU
Cycle 1	Patients assessed, *n*	113	112
	Negative < 1.00, *n* (%)	113 (100.00)	110 (98.21)
	Positive ≥ 1.00, *n* (%)	0	1 (0.89)
	Not analysed, *n* (%)	0	1 (0.89)
Cycle 2	Patients assessed, *n*	111	112
	Negative < 1.00, *n* (%)	111 (100.00)	112 (100.00)
	Positive ≥ 1.00, *n* (%)	0	0
Cycle 4	Patients assessed, n	108	109
	Negative < 1.00, *n* (%)	108 (100.00)	109 (100.00)
	Positive ≥ 1.00, *n* (%)	0	0
Cycle 6	Patients assessed, *n*	108	108
	Negative < 1.00, *n* (%)	108 (100.00)	108 (100.00)
	Positive ≥ 1.00, *n* (%)	0	0
Overall	Patients assessed, *n*	113	112
	Negative < 1.00, *n* (%)	113 (100.00)	111 (99.11)
	Positive ≥ 1.00, *n* (%)	0	1 (0.89)

^a^Percentages are based on the number of patients assessed at each visit. The unit of ADA titre was endpoint titre. Only predose assessments are summarised.

ADA = antidrug antibody; trastuzumab-EU = licensed trastuzumab sourced from the European Union

## Discussion

The primary objective of this comparative clinical trial in patients with operable HER2+ breast cancer was to determine whether neoadjuvant treatment with PF-05280014, a potential trastuzumab biosimilar, was non-inferior to trastuzumab-EU, based on pharmacokinetic data. This objective was met; the study demonstrated non-inferiority for PF-05280014 versus trastuzumab-EU in the percentage of patients with Cycle 5 *C*_trough_ (Cycle 6 predose) > 20 μg/ml. Furthermore, results from an analysis of secondary endpoints demonstrated comparable pharmacokinetic profiles for PF-05280014 and trastuzumab-EU.

PF-05280014 and trastuzumab-EU also had comparable efficacy profiles, based on measures of tumour control, with no notable difference between treatment groups (PF-05280014 vs trastuzumab-EU, respectively) in pCR rate (47.0% vs 50.0%) or ORR (88.1% vs 82.0%). Importantly, pCR results were consistent with published data for trastuzumab administered in combination with neoadjuvant chemotherapy.^27,28^ Furthermore, both pCR and ORR were comparable to rates reported for other proposed trastuzumab biosimilars in development and evaluated in the neoadjuvant setting.^29,30^

Both treatments were generally well tolerated, with few patients discontinuing from any treatment due to AEs. There was no clinically significant imbalance between groups in TEAEs, serious TEAEs or other observed safety parameters. PF-05280014 and trastuzumab-EU also had comparable immunogenicity profiles, wherein no patients in the PF-05280014 group and one patient in the trastuzumab-EU group tested positive for ADAs. These data are consistent with previous studies demonstrating a low immunogenic potential for trastuzumab.^19,31^ As part of a stepwise comparison exercise to demonstrate biosimilarity, these findings build on those of previous analytical and non-clinical studies and two single-dose studies in healthy volunteers.^18–20^

The current study applied a non-inferiority design to compare PF-05280014 with trastuzumab-EU. Generally, biosimilarity studies use an equivalence design to show the biosimilar is neither superior nor inferior to the originator product and vice versa.^16,17,32^ However, in some cases, a non-inferiority design may be adequate to demonstrate that there are no clinically meaningful differences between the proposed biosimilar product and originator biologic.^16^ This study may be limited by its use of a margin for non-inferiority of the clinical endpoint pCR to establish non-inferiority using a primary endpoint based on pharmacokinetics data. However, a non-inferiority margin based on clinical considerations rather than statistical considerations is supported by a lack of previous evidence in the neoadjuvant setting outside of the HannaH trial.^26^ In addition, this study was not powered to evaluate non-inferiority in pCR or ORR, which may limit interpretation of clinical efficacy findings. However, a comparative study of PF-05280014 versus trastuzumab-EU, each in combination with paclitaxel, as first-line treatment in patients with HER2+ metastatic breast cancer used an equivalence design, and preliminary results demonstrated that this trial met its primary endpoint of similarity in ORR.^33^

The strengths of the current trial include its randomised, double-blind design, homogeneous patient population and use of clinical endpoints that measure activity (e.g., tumour response). The most frequently published definition of pCR (absence of invasive neoplastic cells in breast and lymph nodes following neoadjuvant therapy) was used, consistent with previous studies of trastuzumab administered concurrently with chemotherapy in the neoadjuvant setting.^11,12,26^ Furthermore, calculation of ORR for efficacy analysis was based on independent central radiologic review of tumour assessments. Finally, neoadjuvant use of trastuzumab is not currently approved by the US Food and Drug Administration^1^; however, it is authorised by the European Medicines Agency and is well known and used in clinical practice in Europe and the United States.^2,25,34,35^ Therefore, the finding of comparable efficacy in terms of pCR and ORR in this setting may be reassuring for patients and clinicians.

In conclusion, neoadjuvant treatment with the potential biosimilar PF-05280014 administered in combination with docetaxel and carboplatin demonstrated non-inferiority in pharmacokinetics and comparability in efficacy, safety and immunogenicity when compared with trastuzumab-EU in combination with docetaxel and carboplatin in patients with operable HER2+ breast cancer. These results support similarity of PF-05280014 to trastuzumab-EU as part of the stepwise comparison exercise for demonstrating biosimilarity. As a potential biosimilar of trastuzumab, PF-05280014 could broaden the number of treatment options for patients with HER2+ breast cancer and allow greater use of anti-HER2 therapy across clinical settings.

## Electronic supplementary material

Supplementary Information

## Availability of data and materials

Pfizer’s policies on the provision of clinical trial data are set out on our website: http://www.pfizer.com/research/clinical_trials/trial_data_and_results. In addition to posting clinical trial results on the ClinicalTrials.gov registry, Pfizer will provide access to anonymised patient-level data in response to scientifically valid research protocols. Data from Pfizer-sponsored global interventional clinical studies are available from: trials conducted for medicines, vaccines and medical devices for indications that have been approved in the US and/or EU; trials conducted for medicines, vaccines and medical devices that have been terminated (i.e., development for all indications has been discontinued). Data from these trials will be made available 24 months after study completion. Pfizer will make reasonable efforts to fulfil all data requests for legitimate research purposes, but there may be instances in which retrieval or delivery of data is not feasible (for example, if Pfizer does not have legal authority to provide the data, if costs of retrieval of older or pre-electronic data are prohibitive, etc. See page 5* at the following link: https://www.pfizer.com/files/research/research_clinical_trials/A_Guide_to_Requesting_Pfizer_Patient-Level_Clinical_Trial_Data_2017.pdf). Further detail can be found at: http://www.pfizer.com/research/clinical_trials/trial_data_and_results/data_requests. Pfizer’s practices adhere to the principles for responsible data sharing laid out by the European Federation of Pharmaceutical Industries and Associations (EFPIA) and the Pharmaceutical Research and Manufacturers of America (PhRMA): http://phrma.org/sites/default/files/pdf/PhRMAPrinciplesForResponsibleClinicalTrialDataSharing.pdf.
