# Supplementary material for: Neoadjuvant PF-05280014 (a potential trastuzumab biosimilar) versus trastuzumab for operable HER2+ breast cancer
Source: Br J Cancer. 2018 Jul 13;119(3):266–73. doi: 10.1038/s41416-018-0147-1 (PMC6068194; doi:10.1038/s41416-018-0147-1)
Supplement: Supplementary file 1 — Supplementary Information [file 41416_2018_147_MOESM1_ESM.pdf]

**Supplementary Table S1. Predose concentration–time values (per protocol population)[a]**

| Visit | Planned time predose | N | NALQ | Mean (± SD), μg/ml | CV (%) | Median (minimum, maximum), μg/ml |
|---|---|---|---|---|---|---|
| **PF-05280014** | | | | | | |
| Cycle 1/Day 1 | 0 h | 101 | 4 | 2.313 (17.949) | 776 | 0 (0, 170) |
| Cycle 2/Day 21 | 0 h | 99 | 99 | 24.29 (13.796) | 57 | 22.40 (1.51, 131) |
| Cycle 4/Day 63 | 0 h | 98 | 98 | 33.43 (14.488) | 43 | 30.75 (6.19, 110) |
| Cycle 5/Day 84 | 0 h | 101 | 101 | 35.01 (15.571) | 44 | 33.00 (10.5, 123) |
| Cycle 6/Day 105 | 0 h | 101 | 101 | 37.77 (17.523) | 46 | 35.60 (11.0, 129) |
| **Trastuzumab-EU** | | | | | | |
| Cycle 1/Day 1 | 0 h | 88 | 1 | 1.318 (12.366) | 938 | 0 (0, 116) |
| Cycle 2/Day 21 | 0 h | 88 | 88 | 27.20 (10.650) | 39 | 26.30 (7.88, 97.8) |
| Cycle 4/Day 63 | 0 h | 89 | 89 | 37.33 (15.629) | 42 | 33.90 (12.5, 127) |
| Cycle 5/Day 84 | 0 h | 87 | 87 | 40.44 (26.765) | 66 | 35.90 (11.3, 206) |
| Cycle 6/Day 105 | 0 h | 89 | 88 | 40.10 (16.670) | 42 | 39.80 (0, 145) |

[a]Below the lower limit of quantification (0.5 μg/ml), concentration was given a value of 0.
CV=coefficient of variation; N=number of patients; NALQ=number of observations above lower limit of quantification; SD=standard deviation; trastuzumab-EU=licensed trastuzumab sourced from the European Union.

**Supplementary Table S2. 1 h postdose concentration values (per protocol population)[a]**

| Visit | Planned time postdose | N | NALQ | Mean (± SD), µg/ml | CV (%) | Median (minimum, maximum), µg/ml |
|---|---|---|---|---|---|---|
| **PF-05280014** | | | | | | |
| Cycle 1/Day 1 | 1 h | 97 | 96 | 160.4 (57.329) | 36 | 156.0 (0, 452) |
| Cycle 5/Day 84 | 1 h | 90 | 90 | 137.0 (37.748) | 28 | 134.5 (13.5, 245) |
| **Trastuzumab-EU** | | | | | | |
| Cycle 1/Day 1 | 1 h | 80 | 80 | 164.8 (47.033) | 29 | 158.0 (68.7, 357) |
| Cycle 5/Day 84 | 1 h | 80 | 80 | 138.8 (37.417) | 27 | 142.0 (38.7, 236) |

[a]One patient (PF-05280014 group) had a 1 h postdose sample with concentration of 0.
CV=coefficient of variation; N=number of patients; NALQ=number of observations above lower limit of quantification; SD=standard deviation; trastuzumab-EU=licensed trastuzumab sourced from the European Union.

**Supplementary Table S3. Summary of cardiac function evaluation LVEF (safety population)**

| Visit | LVEF | PF-05280014 | Trastuzumab-EU |
|---|---|---|---|
| Screening | Patients assessed, *n* | 113 | 112 |
| | Mean (± SD), % | 65.1 (4.59) | 65.6 (5.28) |
| | Median, % | 65.0 | 65.0 |
| | Range, % | 56–85 | 55–80 |
| Cycle 3 | Patients assessed, *n* | 111 | 110 |
| | Mean (± SD), % | 64.2 (4.83) | 64.9 (5.10) |
| | Median, % | 64.0 | 64.0 |
| | Range, % | 53–81 | 55–78 |
| EOT | Patients assessed, *n* | 110 | 109 |
| | Mean (± SD), % | 64.5 (4.62) | 63.8 (4.52) |
| | Median, % | 64.0 | 63.0 |
| | Range, % | 55–83 | 55–75 |

EOT=end of treatment; LVEF=left ventricular ejection fraction; SD=standard deviation; trastuzumab-EU=licensed trastuzumab sourced from the European Union.
